# Catarrhal Urethritis in Boys

**Published:** 1894-08

**Authors:** 


CATARRHAL URETHRITIS IN BOYS.—Dr. S. Rona, of Buda-pest points out (*Med. Bull.*) that under the title catarrhal urethritis is described as an acute, typical inflammation of the urethra occurring in boys.

It is attributed to various mechanical and chemical irritants, as anemia, introduction of foreign bodies, the action of uric acid crystals, kidney-sand, and larger concretions passing from the bladder through the urethra, an excessive amount of uric acid dissolved in urine, purulent balanitis, itching of the skin in the neighborhood of the pubes, etc. Dr. Rona reports sixteen cases which he had observed, and concludes that the inflammation, at least in the majority of cases, is of gonorrheal origin, and should be regarded as genuine gonorrhea. This opinion had already been advanced by Cseri, who had found gonococci in the discharge. The whole course of the affection, which often lasts for months, closely resembles that of the gonorrhea of adults. The same is true of the complications, such as balano-posthitis, lymphadenitis, lymphangitis of the penis, cystitis, and epididymitis, although these are less frequent in boys than in men. Dr. Rona saw developed, in a fifteenth-month old babe, a typical double-sided orchitis, which required several weeks for its cure. Once he met with gonorrhea in two girls and boys of the same family. In all cases gonococci were recognized in the discharge. From these researches it follows that the urethritis of children should not be lightly regarded, and that its prognosis is much more grave than is generally supposed. As complications, gonorrheal conjunctivitis was twice observed, and in one case had a favorable termination. The course of the affection was very slow, being prolonged, on an average, for two or three months, and some cases continued for a half or three-fourths of a year. The six cases of non-gonorrheal origin exhibited no peculiarity. In some the discharge was tenacious, grayish white, and scanty; in others a grayish yellow thin pus was observed. Two cases were relics of a gonorrhea of the previous year. Others resulted from the propagation of suppuration from surrounding parts to the vulva. Finally, a form was seen in new-born babes attributable to severe catarrh of the desquamative genital mucous membrane.—*Medical Standard. Ex.*

